# Research Progress on the Biological Function, Disease-Driving Mechanism and Clinical Targeting Strategies of G3BP2

**DOI:** 10.3390/molecules31040622

**Published:** 2026-02-10

**Authors:** Yao Chen, Qi Deng, Li-Ling Yang, Ai-Ling Jiang, Rong Zhang, Qi-Bing Yan, Yong-Kang Wu

**Affiliations:** 1 Emergency Department, West China Hospital Sichuan University Jintang Hospital, Jintang First People’s Hospital, Chengdu 610400, China; 2 Department of Gastroenterology, West China Hospital Sichuan University Jintang Hospital, Jintang First People’s Hospital, Chengdu 610400, China; 3 Nursing Department, West China Hospital Sichuan University Jintang Hospital, Jintang First People’s Hospital, Chengdu 610400, China; 4 Department of Laboratory Medicine, West China Hospital Sichuan University Jintang Hospital, Jintang First People’s Hospital, Chengdu 610400, China; 5 Department of Laboratory Medicine, West China Hospital, Sichuan University, Chengdu 610065, China

**Keywords:** G3BP2, stress granules, breast cancer, targeted therapy

## Abstract

G3BP2 is an important RNA-binding protein that belongs to the mammalian Ras-GAP SH3 domain-binding protein (G3BP) family. Its structure enables it to bind to RNA or proteins, regulate nuclear–cytoplasmic shuttling, and participate in various functions, including cell growth, differentiation, migration, and RNA and protein metabolism. Studies have found that G3BP2 is involved in the occurrence and development of various human diseases, such as high expression across multiple tumor diseases, including gastric cancer, breast cancer, non-small-cell lung cancer, esophageal squamous cell carcinoma, colorectal cancer, and pancreatic ductal adenocarcinoma, driving the occurrence of human tumors, participating in tumor progression, and playing an essential role in promoting the proliferation, invasion, and migration of tumor cells. Additionally, G3BP2 is closely associated with various non-tumor diseases, including viral infections, as well as cardiovascular and cerebrovascular diseases. This review elucidates the role of G3BP2 in the development and progression of various diseases, identifying biomarkers and therapeutic targets for clinical diagnosis and treatment based on G3BP2.

## 1. Structure, Expression, Regulation, and Mechanism of G3BP2

### 1.1. Structure of G3BP2

**Figure 1:**
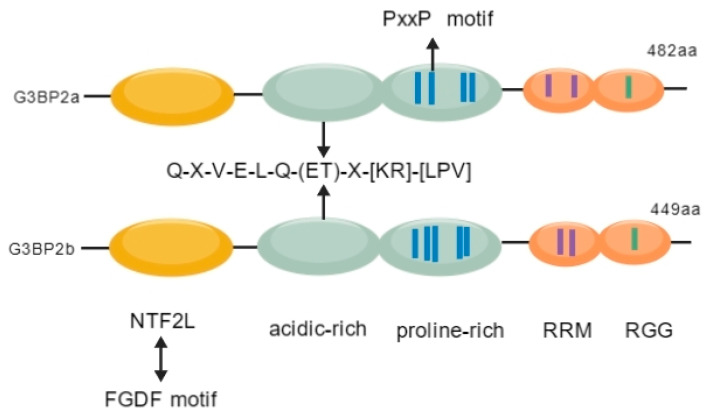
**The structure of G3BP2.** The distribution of the domains of G3BP2a and G3BP2b is shown: The N-terminal NTF2L is on the left, the central disordered region contains phosphorylation sites (pS) and the PxxP motif, and the C-terminal RBD binds to RNA and contains the RGG repeat sequence. The overall presentation shows their modular structure and molecular interactions.

G3BP2 is an important RNA-binding protein in the mammalian Ras-GAP SH3 domain-binding protein (G3BP) family. Its structure supports dual RNA–protein binding, thereby regulating nuclear–cytoplasmic shuttling, cell growth, differentiation, migration, and RNA/protein metabolism functions [1,2]. This protein was first isolated and named by D. Kennedy et al. from the Mmu-G3B protein in 1997. Abnormal activation of this pathway is associated with multiple pathologies. G3BP2 consists of two splicing isoforms: G3BP2a (482 amino acids, 68.5 kDa) and G3BP2b (449 amino acids, 62 kDa). The difference between the two is that G3BP2b lacks 33 amino acids in the central region of the primary structure [3]. This protein exhibits a highly modular structure, with the N-terminal part comprising the NTF2L domain [4]. The central intrinsic disordered region is further divided into acidic-rich domains containing the conserved protein-binding motif Q-X-V-X-E-L-Q-(ET)-X-[KR]-[LPV], which is also found in interacting proteins such as IκBα and MDM2 [5], and contains proline-rich domains (G3BP2a has four PxxP motifs, and G3BP2b has five) [3]. The C-terminal region comprises the RNA-binding domain (RBD), which mediates RNA-specific binding and transcript stability [6,7], and disordered fragments containing RGG repeat sequences, which synergistically enhance RNA binding [8] (Figure 1).

### 1.2. Expression Regulation of G3BP2

#### 1.2.1. Transcriptional Regulation of G3BP2

G3BP2 activates gene transcription in prostate cancer (PCa) cells [9,10]. The promoter region (from −740 to −734) contains the Foxd1 binding sequence GTAAACA [11]. Androgens and Foxd1 participate in the p53 pathway by regulating G3BP2 [12].

#### 1.2.2. Translational Regulation of G3BP2

In patients with diabetes and cells exposed to high glucose levels, G3BP2 activates p38 MAPK/p53 to inhibit the miR-23b signaling pathway, forming a miR-23b/G3BP2 feedback loop that regulates p38 MAPK/p53 [13,14]. Meanwhile, the p38MAPK signaling pathway can promote G3BP2 expression and mediate the functional impairment of renal endothelial cells induced by high glucose levels [15]. This regulatory axis is also present in osteosarcoma and is a key mechanism of its malignant progression [16]. Furthermore, the study hypothesizes that the circFNDC3B/miR-1178-3p/G3BP2 signaling axis may be involved in the migration and invasion of bladder cancer [17,18].

#### 1.2.3. Post-Translational Modification

##### Phosphorylation

The G3BP protein contains multiple conserved phosphorylation sites. Serine residues 149 and 232 are highly conserved between G3BP1 and G3BP2 [19]. RIOK1 enhances the activity of G3BP2 by phosphorylating Thr226 [20]. Furthermore, G3BP2 can regulate the phosphorylation of LRP6 and the classical Wnt-β-catenin signaling pathway [21]. Through LYN-mediated phosphorylation of TWIST1, TWIST1 dissociates from G3BP2 and enters the nucleus, driving epithelial–mesenchymal transition (EMT) and invasion [22]. PKCα can phosphorylate G3BP2 in vitro and co-localize with it in stress granules (SGs) [23]. Cells lacking G3BP1/2 lose the ability to form SGs when eIF2α is phosphorylated or eIF4A is inhibited, but can still form SGs in response to high temperature or osmotic pressure [24], and eliminate the inhibition of TRBP on the phosphorylation of IRF-3 [25]. Under irradiation, the G3BP2-ROBO1-eIF3A complex triggers lysosomal protein degradation, and G3BP2 knockout inhibits ROBO1 regulation on the stability of eIF3A [26].

##### Polyubiquitination

G3BP2 recruits the deubiquitinating enzyme USP10, which binds through its N-terminal and central region 140-252aa, and USP10 maintains the stability of G3BP2 by reducing its polyubiquitination [27]. LINC2781 directly binds to G3BP2, thereby blocking the degradation of STAT1 mediated by G3BP2 [28]. G3BP2 also interacts with hnRNPA2B1 [29].

##### Arginine Methylation

The RGG domain of G3BP2 serves as a methylation target for PRMTs, downstream of the Wnt3a signaling pathway. Methylated G3BP2 binds to Dvl3, forming a complex centered on Dvl3 that includes methylated G3BP2, kinase CKI, and GSK3 [21].

#### 1.2.4. Regulation of G3BP2 Subcellular Localization

G3BP2 is primarily found in the cytoplasm, with a small amount in the nucleus, and its location is closely related to its function. The absence of G3BP2, a core component of autophagosome assembly, significantly weakens cancer cell proliferation and migration when environmental stress inhibits autophagy [30].

### 1.3. Mechanism of G3BP2

G3BP2 is abnormally expressed in lung cancer, breast cancer (BC), PCa, and diseases, including cardiac hypertrophy and atherosclerosis, and is a potential therapeutic target [12].

#### 1.3.1. RNA Binding-Mediated Transcript Stability

G3BP2 stabilizes SART3 mRNA (for T-cell targeting) by combining the target RNA to regulate its stability with hepatocellular carcinoma (HCC)-derived growth factor (HDGF) mRNA [12,31]. HDGF is highly expressed in cancer and promotes metastasis by activating EMT signaling, actin reorganization, and matrix adhesion. G3BP2 regulates its expression [12,32,33].

#### 1.3.2. Regulation of Protein Subcellular Localization

**Figure 2:**
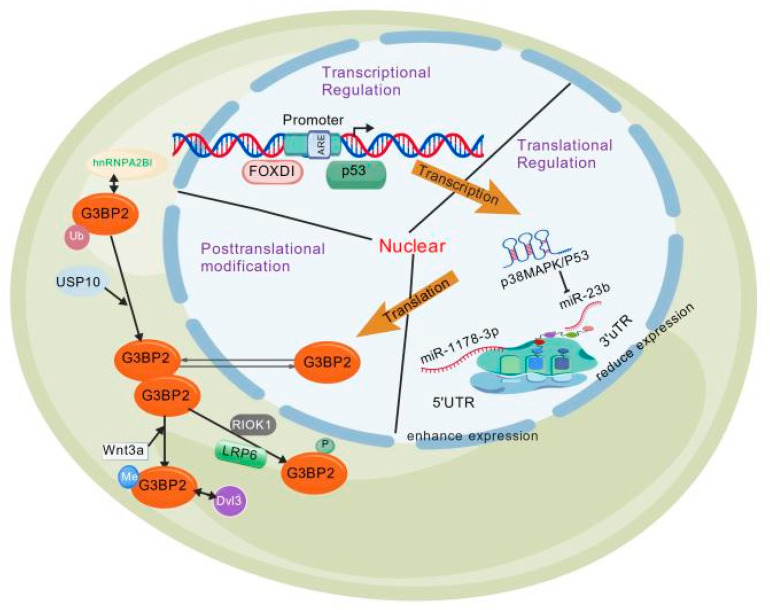
**The expression regulation of G3BP2.** shows G3BP2’s multi-level regulation. In the nucleus, FOXO3/p53 regulates transcription via the Foxd1 site. In the cytoplasm, miR-23b controls translation; high glucose triggers p38MAPK feedback. Post-translation, USP10 deubiquitinates, kinases phosphorylate, and PRMTs methylate. Mainly cytoplasmic, its function ties to the subcellular location.

G3BP2 is upregulated in hypertrophic myocardial tissues, where it interacts with IκBα to promote nuclear accumulation of p65, thereby enhancing the transcriptional activity of NF-κB and driving hypertrophy [34,35]. Additionally, the acidic/proline-rich domain and RRM domain of G3BP2 mediate its binding to IκBα CRS, while the NTF2L/RBD promotes its retention in the cytoplasm [36]. Furthermore, G3BP2 is involved in regulating the p53 pathway. G3BP2 binds to cytoplasmic p53, thereby increasing its cytoplasmic localization [37]. Combining the RING domain of MDM2 enhances the monoubiquitination modification and degradation of p53 by MDM2, stabilizes the expression of MDM2/p53, and promotes the nuclear export of p53 [38]. Androgens promote nuclear export and SUMOylation of p53 through the G3BP2-RanBP2 interaction in PCa [9,39,40]. TRIM25 forms a complex with G3BP2/RanBP2 in 22Rv1 cells [41] (Figure 2).

#### 1.3.3. Cytosolic Localization Regulation of TWIST1

Tyr10 phosphorylation disrupts the binding motif of G3BP2, releasing TWIST1 into the nucleus to drive BC EMT [5]. α-Parvin binds to G3BP2 through its N-terminal sequence (V-S-E-L-Q-E), blocking its interaction with TWIST1, inhibiting the ubiquitination and degradation of TWIST1, and promoting its nuclear import [42,43]. The RGG domain of G3BP binds to the lysosomal TSC complex, and through the NTF2L domain, it bridges TSC2 to LAMP2, thereby inhibiting the mTORC1 signal before SG formation [44,45].

#### 1.3.4. Core Role of SG Assembly

G3BP2 is a core component of SG [44,46] and can independently mediate SG assembly [19]. The arginine residues in the RGG domain are prone to oligomerization and efficiently form RNA condensates [19,47,48]. Additionally, SGs are regulated by the carcinogenic protein G3BP2 [30]. G3BP2 participates in the specific binding of NCAP and is transported to SGs after oxidative stress or heat shock [49]. Furthermore, the research has identified the following regulatory pathways. PKCα phosphorylates the NTF2L domain of G3BP2 (neuroblastoma) [23]. UBAP2L binds to the G3BP2-NTF2L domain and retains it in the cytoplasm, participating in the formation of SGs and altering their dynamics by delaying reaggregation [47,50]. The FGDF motif protein (USP10/ICP8/nsP3) binds to G3BP2 to inhibit SG assembly [49]. Mage-b2 inhibits the formation of SGs by interfering with the translation of G3BP2 [51]. The miR-206 inhibits G3BP2 and promotes viral replication [52].

#### 1.3.5. The Relationship Between G3BP1 and G3BP2

G3BP1 and G3BP2 belong to the same G3BP protein family. They share a high degree of similarity in amino acid sequences and protein structures, but their biological functions are significantly different [53]. Both G3BP1 and G3BP2 contain an N-terminal NTF2-like domain, a central disordered region, and a C-terminal RBD. The most significant structural difference lies in the number of PxxP motifs: G3BP1 has 2, G3BP2a has 4, and G3BP2b has 5. In addition, both G3BP1 and G3BP2 bind to multi-nucleolar ribonucleoproteins (mRNP) and regulate translation initiation as well as drive the formation of stress granules (SGs) [54,55]. The other two are located on different chromosomes. G3BP1 is located in the region from 5q14.2 to 5q33.3, while G3BP2 is distributed between 4q12 and 4q24. Both have 65% sequence similarity and 74% structural similarity [3,56]. There are differences in cancer etiology between the two. G3BP1 promotes cell proliferation and invasion by regulating the transcriptional transcripts of PMP22 and BART [57,58,59]. And G3BP2 regulates the cellular localization of Twist1, playing a role in cancer metastasis [5]. In addition, both have tissue-specific expression characteristics. G3BP1 is highly expressed in the lungs, kidneys and colon, while it is minimally expressed in the heart, liver and spleen. G3BP2 is widely distributed, and its subtypes have specificities: G3BP2a is restricted to the brain, muscles and heart, while G3BP2b is only found in the small intestine [12].

## 2. Disease Expression of G3BP2 (Table 1)

### 2.1. Expression of G3BP2 in Tumors

#### 2.1.1. Esophageal Squamous Cell

Esophageal cancer (EC) has a high degree of malignancy and poor prognosis, with a five-year survival rate of 15–25% [60]. Among them, esophageal squamous cell carcinoma (ESCC) is the primary pathological type [61]. Currently, the primary treatment methods for ESCC include surgery, radiotherapy, chemotherapy, targeted therapy, and immunotherapy. The selection of treatment plans should be based on the patient’s clinical stage. High G3BP2 expression is significantly associated with lymph node metastasis, deep tumor invasion, and adverse clinical outcomes. Functional experiments confirmed that G3BP2 enhanced the migration and invasion abilities of ESCC cells. Compound C108 inhibits the migration/invasion of ESCC cells in vitro and blocks tumor metastasis in the in vivo model. Targeting the inhibition of G3BP2, such as C108, is a potential anti-metastatic strategy [34]. G3BP2 forms a feedback loop with BAALC-AS1 and c-Myc, playing a crucial role in ESCC development and providing new targets for treatment [62].

#### 2.1.2. Gastric Cancer (GC)

GC is a highly prevalent malignant tumor worldwide and is the leading cause of cancer-related deaths [63]. An additional one million cases are reported each year, with over 650,000 deaths [64]. The prognosis of patients is significantly correlated with the stage: the five-year survival rate in the localized stage is >70%, while it is <10% in the metastatic stage [65]. In GC, the upregulation of G3BP2 expression is significantly and positively correlated with disease severity [66]. The long non-coding RNA (lncRNA) TM4SF1-AS1 promotes the formation of SG by isolating RACK1 (an activator of the stress MAPK pathway) and inhibits the apoptosis of GC cells. Its effect is associated with SG proteins, such as G3BP2 [67]. The expression levels of RABEP2 and G3BP2 in the gastric epithelium gradually increase from precancerous lesions (intestinal metaplasia and atypical hyperplasia) to GC progression. This is significantly higher than that in gastritis tissues, suggesting that the protein changes induced by *Helicobacter pylori* promote the occurrence of GC [66].

#### 2.1.3. Colorectal Cancer (CRC)

CRC is the third most common cause of cancer-related deaths worldwide, with over 1.85 million new cases and 850,000 deaths annually. Approximately 20% of patients have already experienced metastasis at the time of initial diagnosis, and another 25% of those initially diagnosed with localized lesions will develop metastasis later [68]. Treatment options include endoscopic or surgical local resection, neoadjuvant radiotherapy and chemotherapy, local ablation therapy, and palliative measures such as chemotherapy, targeted therapy, and immunotherapy [69]. Studies have revealed that G3BP2 is a key factor in the proliferation, migration, and maintenance of stem cells in CRC. Its absence can inhibit these malignant phenotypes, while hsa_circRNA_001676 promotes the proliferation, migration, and stem cell progression of CRC by regulating the miR-556-3p/G3BP2 axis [70].

#### 2.1.4. Pancreatic Ductal Adenocarcinoma (PDAC)

PDAC accounts for >90% of pancreatic cancer cases and is the seventh leading cause of cancer-related death worldwide. The primary treatment approach includes surgical resection combined with systemic chemotherapy (sometimes supplemented by radiotherapy), which is currently the only potentially curative treatment. Nevertheless, the long-term prognosis of patients remains poor, even with improved chemotherapy regimens [71]. At the molecular level, DKC1 (involved in RNA stability regulation) is a target of G3BP2. G3BP2 binds to PDIA3 mRNA and recruits it to SGs, thereby enhancing the stability of PDIA3 mRNA while reducing its translation efficiency, thereby promoting DKC1 expression of DKC1 [72].

#### 2.1.5. Hepatocellular Carcinoma (HCC)

Liver cancer, especially HCC, which accounts for approximately 90% of all cases, is a major global health burden. The number of new cases is projected to exceed one million by 2025. HCC is mainly associated with aflatoxins, hepatitis B virus, hepatitis C virus, and metabolic disorders [73]. Radical treatments (surgical resection, ablation, or transplantation) are available for early-stage HCC. For middle-stage HCC, local regional treatments are primarily adopted; for advanced-stage HCC, the preferred treatment option is based on immune checkpoint inhibitors [74]. Furthermore, ANCCA/PRO2000 enhances the migration ability of HCC cells to a certain extent by inhibiting the expression of ERO1L and G3BP2 [75].

#### 2.1.6. Breast Cancer (BC)

**Figure 3:**
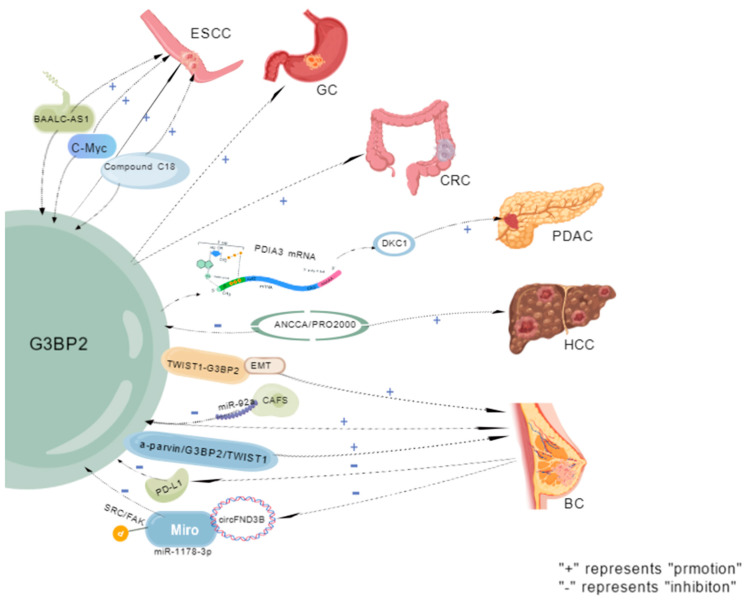
**Disease expression of G3BP2.** The figure illustrates the oncogenic role of G3BP2 in various cancers. It is located in the center and promotes tumor progression through pathways such as BAALC-AS1/c-Myc (ESCC), PDIA3 mRNA (GC), DKC1 (CRC), ANCCA (PDAC), TWIST1/EMT (HCC), miR-92a/PD-L1/SRC-FAK (BC). Most of these are indicated by a “+” sign, suggesting that G3BP2 is a potential pan-cancer therapeutic target.

BC exhibits the highest incidence and mortality rates among malignant tumors in women globally, with both figures continuing to rise. The application of deep learning technology has significantly improved its diagnostic accuracy and efficiency, especially in predicting metastasis and assessing prognosis [76], as well as in immunotherapy [77]. Studies have demonstrated that G3BP2 plays multiple roles in BC its high expression enhances cell stemness, metastatic ability, and chemotherapy resistance [31,78], and it drives tumor occurrence by stabilizing SART3 mRNA and upregulating the expression of pluripotency factors Oct-4/Nanog [31]. Microenvironment response: The arrangement of collagen (a marker of matrix hardness) and low expression of G3BP2 jointly indicate poor prognosis. The mechanical transduction pathway of TWIST1-G3BP2 can respond to microenvironmental signals, inducing EMT and metastasis [5]. The contradictory role of G3BP2 in BC is that it acts as an inhibitor of cell migration/invasion, while miR-92a, derived from CAFs, promotes this process by downregulating G3BP2 expression [79]. The synergistic regulatory effect is manifested in the formation of a new metastasis-promoting pathway involving α-parvin/G3BP2/TWIST1, whose activation is a key driving factor for the progression of ER-negative BC [43]. Additionally, under chemical stress, G3BP2 and PD-L1 synergistically upregulate, and inhibition of G3BP2 can reduce PD-L1 expression [80]. Moreover, its expression is regulated at multiple levels: SK1 knockout downregulates NSUN2, NFATC3, CDK2, and G3BP2 [81]. The circBACH1/miR-217 axis (paclitaxel-induced exosomal circBACH1 sequesters miR-217 through sponge adsorption to relieve its inhibition of G3BP2) [82]. The circFNDC3B overexpression inhibits G3BP2 by adsorbing miR-1178-3p, blocking the phosphorylation of SRC/FAK pathways, thereby inhibiting the malignant behavior of BC [83] (Figure 3):

#### 2.1.7. Non-Small-Cell Lung Cancer (NSCLC)

NSCLC is the most common and fatal malignant tumor. Current treatment strategies for non-metastatic patients include perioperative combined adjuvant immunotherapy to increase the cure rates. For patients with metastasis, new drugs are used to prolong disease control time and maintain quality of life [84]. One of the three core regulatory mechanisms of G3BP2 in NSCLC involves nuclear localization. The TRIM domain interacts with G3BP2, promoting its nuclear localization and significantly increasing its nuclear expression, which can inhibit the proliferation, migration, and invasion of H1299 cells [30]. The second core regulatory mechanism involves the stress response. Under ETV4-induced low lactate stress, G3BP2 is selectively translated, inhibiting the activity of mTORC1 by recruiting the lysosome-TSC2 complex, thereby becoming a key component in regulating glycolysis and protein synthesis through the ETV4/TSC2/mTORC1 axis [85]. The third core regulatory mechanism involves regulating sensitivity to treatment. Recombinant human MG53 (rhMG53) induces the nuclear translation of G3BP2 and blocks the formation of SGs, inhibiting the proliferation of NSCLC cells and enhancing cisplatin sensitivity [30].

#### 2.1.8. PCa

PCa is a major global health burden, causing over 375,000 male deaths annually [86]. Current primary treatment methods, such as radical prostatectomy, radiotherapy, and ablation therapy, have limited effects [87]. TRIM25 overexpression regulates p53 nuclear output by interacting with G3BP2, thereby promoting cancer cell proliferation and survival [88]. G3BP2 is a direct target gene of the androgen receptor (AR) and is driven by androgens to translocate to the cytoplasm via p53. It exerts a cancer-promoting effect by advancing the cell cycle and inhibiting apoptosis. The nucleoplasmic transport of p53 is mediated by RanBP2 (SUMO-E3 ligase), which interacts with G3BP2, regulated by androgen-dependent SUMOylation. In the androgen deprivation therapy model, G3BP2 knockdown inhibited tumor growth and increased nuclear p53 accumulation. Clinical studies have revealed that the strong localization of cytoplasmic p53 is significantly associated with a high expression of G3BP2, suggesting a poor prognosis and an increased risk of hormone-refractory transformation [9].

### 2.2. Expression of G3BP2 in Viral Infections

Viruses are obligatory intracellular parasites that use host cell mechanisms to synthesize their proteins [89]. G3BP2 exerts an antiviral effect by isolating viral proteins from SGs [90].

#### 2.2.1. SARS-CoV-2

SARS-CoV-2 is a positive-sense single-stranded virus belonging to the beta coronavirus genus [91]. SARS-CoV-2 infection can cause symptoms such as fever, cough, chest discomfort, and viral pneumonia, including breathing difficulties and bilateral lung infiltration [92]. G3BP1/G3BP2 (RAS GTPase-activating protein SH3 domain-binding protein 1/2) is a key host factor that interacts with the SARS-CoV-2 nucleocapsid (N) protein. Their binding disrupts SG formation. Knockout of either G3BP1 or G3BP2 can enhance SARS-CoV-2 replication, indicating that they play crucial roles in regulating the host–virus interface during infection [93].

#### 2.2.2. Chikungunya Virus (CHIKV)

CHIKV is a single-stranded positive-sense RNA virus belonging to the Togaviridae family and transmitted by mosquitoes. It can cause widespread outbreaks of viral arthritis and has led to multiple epidemics worldwide, posing a significant threat to public health [94]. CHIKV infection presents as an acute febrile disease [95]. The simultaneous absence of G3BP1 and G3BP2 in the CHIKV reduces the RNA level, protein expression, and titer of the progeny, indicating that the CHIKV also relies on G3BPs for efficient replication. Studies show that G3BP1 knockdown does not impair CHIKV replication—likely due to compensatory upregulation of G3BP2. G3BP2 co-localizes with nsP2 and nsP3 (but not nsP1, nsP4, or dsRNA) in cytoplasmic viral particles [96]. CHIKV replicon or nsP3 alone recruits G3BP1; during late infection, G3BP1 and G3BP2 co-localize in virus-induced particles—distinct from canonical SGs in morphology, CHX sensitivity, and composition, indicating virus-specific assemblies [97].

#### 2.2.3. Foot-and-Mouth Disease Virus

Foot-and-mouth disease is a highly contagious animal disease caused by the foot-and-mouth disease virus. It is widely prevalent in Asia and can infect various domestic and wild herbivorous animals [98]. Typical symptoms include fever and a limp. Painful blisters appear in the oral cavity and hairless skin areas, such as the crown area, between the toes, and on the nipples, accompanied by increased salivation [99]. It can antagonize SG formation by targeting the scaffold proteins G3BP1 and G3BP2 and cleaving these proteins with its L proteinase. It regulates the comprehensive stress response to evade the host antiviral defense. This will aid in formulating control strategies for FMDV infections in the future [100].

#### 2.2.4. Dengue Virus (DENV)

DENV is a Flavivirus with positive-strand (+) single-stranded RNA characteristics. The enveloped virus particles are spherical, with their surface proteins arranged in icosahedral symmetry. It is the primary host of human dengue fever, which is transmitted by mosquitoes of the genus Aedes [101]. DENV RNA can bind to regulatory proteins of P bodies (PB)/SG, such as DDX6, G3BP1, G3BP2, Caprin1, and USP10. Among them, the SG-related proteins (G3BP1, G3BP2, Caprin1, and USP10) specifically bind to the variable region of the 3′ untranslated region (UTR) of DENV RNA. These results suggest that the 3′ UTR of DENV-2 serves as an assembly platform for PB/SG-related proteins, and assembly on the 3′ UTR is necessary for viral replication [102].

### 2.3. G3BP2 Expression in Cardiovascular and Cerebrovascular Diseases

#### 2.3.1. Myocardial Hypertrophy

Cardiac hypertrophy, defined as the absolute increase in ventricular mass, is among the most robust markers of increased risk for developing HF, independently of the underlying cause [103,104]. Cardiac hypertrophy manifests clinically across diverse pathologies, including sustained pressure/volume overload, ischemic disease, and genetic disorders [105]. The associated myocardial remodeling can lead to heart failure, which is one of the main mechanisms of morbidity and mortality in the elderly [106]. Research has found lnc9456 promotes the nuclear translocation of NF-κB through physical interaction with G3BP2, thereby activating the hypertrophy-related cascade reaction [107]. The hypertrophy responses of neonatal rat cardiomyocytes (NRCMs) induced by overexpression of G3BP2 or isoproterenol (ISO) can be significantly inhibited by NF-κB inhibitors PDTC (50 μmol/L) or p65 knockdown. These results provide new insights into the mechanism of G3BP2’s role in myocardial hypertrophy [35].

#### 2.3.2. Damage to Retinal Microvascular Endothelial Cells in the Human Eye

Diabetic retinopathy (DR), a leading microvascular complication of diabetes mellitus, features retinal aneurysms, hemorrhage, and—progressively—neovascularization and fibroplasia, which may cause severe vision loss or blindness. Globally, ~103 million people had DR in 2020; prevalence is projected to rise to 160.5 million by 2045 [108]. DR progresses through two main stages: non-proliferative DR (NPDR), marked by microaneurysms, retinal hemorrhages, hard exudates, and cotton-wool spots—reflecting early vascular and neuronal injury; and proliferative DR (PDR), defined by pathological neovascularization, vitreous hemorrhage, and fibrovascular proliferation due to severe hypoxia and VEGF-driven angiogenesis. Anti-VEGF therapy is the mainstay pharmacologic treatment but is primarily reserved for advanced disease (e.g., PDR or DME) [109]. Research has found that lncRNA TDRG1 upregulates the transcription level of G3BP2 by competitively binding to miR-7-5p, thereby exacerbating damage to human retinal microvascular endothelial cells (hRMECs) induced by high glucose [110].

#### 2.3.3. Alzheimer’s Disease (AD)

Alzheimer’s disease is a leading cause of senile dementia worldwide. It is an incurable progressive condition that typically leads to patient mortality [111]. Alzheimer’s disease currently affects about 55 million people worldwide, and its prevalence doubles every five years [112]. Research has foundG3BP2 directly binds to the tau protein and inhibits its pathological aggregation. This interaction is significantly enhanced in various tau pathologies in humans, including AD, and is independent of neurofibrillary tangle formation in AD. Mechanistic studies have demonstrated that G3BP2 inhibits tau aggregation by blocking the microtubule-binding region (MTBR). Human neuronal and brain organoid experiments have confirmed that G3BP2 deficiency significantly exacerbates the tau pathological process [46,113].

### 2.4. G3BP2 Expression in Other Diseases

**Figure 4:**
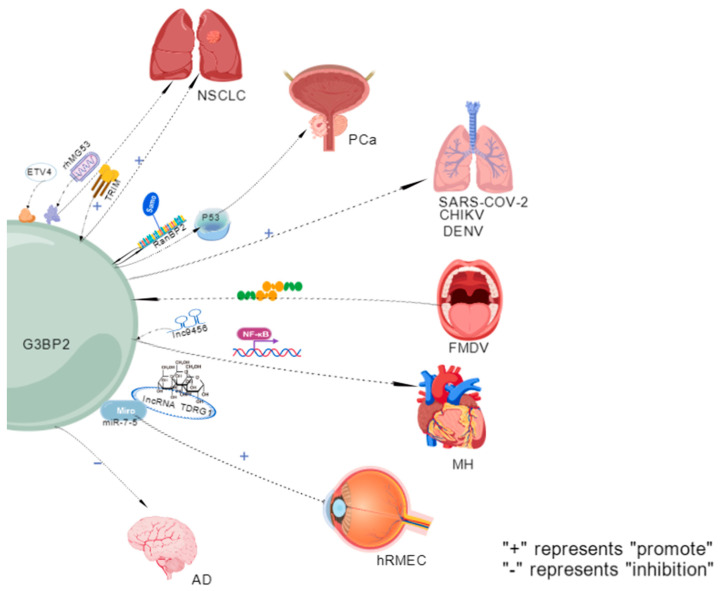
**Disease expression of G3BP2.** G3BP2 plays a crucial regulatory role in various diseases. By interacting with molecules such as P53, lncRNAs (such as hMGA33, Inc9456) and miRNAs, it participates in the progression of diseases such as non-small-cell lung cancer, prostate cancer, and Alzheimer’s disease, and affects the host cell response in various viral infections such as SARS-CoV-2. Abnormal expression of G3BP2 may promote tumor formation or neurodegeneration, suggesting that G3BP2 is an important node molecule in multiple pathological processes.

G3BP2 is involved in immune responses, mRNA transport, and stress granule assembly, and is indispensable for mouse spermatogenesis and male fertility [114]. Additionally, the interaction between PGRMC1/PGRMC2 and G3BP2 dynamically regulates the rate at which SIGCs enter the cell cycle [115]. Studies have also found that G3BP2 promotes psoriasis development by increasing the proportion of CD8+ T cells [116]. miR-363 is indispensable for M1 macrophage polarization and can be released from M1 macrophages through exosomes to induce chondrocyte apoptosis and inflammation. The G3BP2 protein, a functional target of miR-363, was knocked down (simulating the inhibition of miR-363) to reproduce the damaging effects of miR-363 overexpression on chondrocytes. Forcibly increasing the expression of G3BP2 protein significantly weakened the apoptosis and inflammatory response in chondrocytes induced by miR-363. In summary, miR-363 mediates chondrocyte damage by targeting and inhibiting the G3BP2 protein, which is expected to be an essential target for preventing cartilage degeneration and osteoarthritis (OA) development [117] (Figure 4).

**Table 1:** Disease expression of G3BP2.

Classification	Disease	Pathway	Mechanism	Is There any Clinical Sample Verification?	Cell Line /Animals	Result	References
Tumor	Esophageal squamous cell carcinoma (SCC)	Long non-coding RNAlncRNA LINC01554	LINC01554 stabilizes G3BP2 by inhibiting its ubiquitination degradation pathway, which forms a feedback loop to maintain its continuous high expression in ESCC.	Yes	Both	This markedly enhances ESCC cell migration and invasion, driving tumor progression.	[34,62]
		Together with c-Myc and BAALC-AS1, forms a feedback loop				It may also provide novel therapeutic targets for ESCC and facilitate the development of new treatment strategies.	
	Gastric cancer (GC)	TM4SF1-AS1 is associated with multiple stress granule (SG) associated-related proteins, including G3BP2.	SG formation is promoted by sequestering RACK1, a stress-responsive MAPK pathway activator, in GC cells.	Yes	Cell line	G3BP2 upregulation correlates significantly with disease severity; carcinogenic pathway-altered protein subgroups may drive human gastric cancer (GC) progression.	[66,67]
			Helicobacter pylori infection induces distinct gastric proteome alterations.				
	Colorectal cancer (CRC)	hsa_circRNA_001676 regulates; The miR-556-3p/G3BP2 axis	Accelerate the proliferation, migration and stem cell-like transformation of CRC	Yes	Cell line	G3BP2 is a key factor for CRC cell Since the content in cell is too long, we added horizontal line for each row, please confirm. proliferation, migration and stemness maintenance; its depletion inhibits these malignant phenotypes.	[70]
	Pancreatic ductal adenocarcinoma (PDAC)	G3BP2 binds to PDIA3 mRNA and recruits it to stress granules.	Promotes DKC1 expression by enhancing its mRNA stability and reducing translation efficiency.	Yes	Cell line	-	[72]
	Hepatocellular carcinoma (HCC)	ANCCA/PRO2000	Inhibits ERO1L and G3BP2 expression.	Yes	Cell line	Partially enhances hepatocellular carcinoma (HCC) cell migration.	[74,75]
	Breast cancer (BC)	TWIST1-G3BP2 mechanical transduction.	Drives EMT, invasion and metastasis by responding to tumor microenvironment signals.	Yes	Both	Regulates EMT, invasion and metastasis.	[5,31,43,79,80,81,82,83]
		Exosomes derived from Cafa contain miR-92a, which reduces the expression of G3BP2.	Promotes breast cancer cell migration and invasion.			SART3 mRNA stable upregulation enhances pluripotency factor Oct-4/Nanog expression, driving tumorigenesis; it markedly inhibits breast cancer (BC) cell proliferation, migration and invasion.	
		Excessive expression of the circBACH1/miR-217 axis.	The miR-217 directly targets and represses G3BP2, while PTX-induced exosomal circBACH1 interacts with miR-217 to abrogate its suppression of G3BP2 and upregulate G3BP2 expression.			Inhibits BC malignant phenotypes	
		circFNDC3B sequesters miR-1178-3p through sponge adsorption.	Inhibits G3BP2 expression, thereby blocking SRC/FAK signaling pathway phosphorylation.			-	
	Non-small-cell lung cancer (NSCLC)	-	TRIM domain interacts with G3BP2.	Yes	Both	Potentially inhibits H1299 cell proliferation, migration and invasion	[30,85]
			Recruits the lysosome-TSC2 complex to inhibit mTORC1 activity			ETV4/TSC2/mTORC1 axis regulates key glycolysis and protein synthesis components	
			Recombinant human MG53 (rhMG53) induces G3BP2 nuclear translocation and inhibits stress granule formation.			Inhibits NSCLC cell proliferation and enhances cellular sensitivity to cisplatin.	
	Prostate cancer (PCa)	-	TRIM25 overexpression regulates p53 nuclear export by interacting with G3BP2.	Yes	Both	It promotes the proliferation and survival of cancer cells.	[9,88]
			Knockdown of G3BP2.			Inhibits tumor growth and increases p53 accumulation in the nucleus.	
			Driven by androgens, it translocates to the cytoplasm via p53.			Thereby indicating a risk of poor prognosis and hormone-resistant transformation.	
Viral infection	Infection with SARS-CoV-2	G3BP1/G3BP2 (RAS GTPase-activating protein SH3 domain-binding protein 1/2) is a key host factor that interacts with the nucleocapsid (N) protein of SARS-CoV-2.	G3BP2 is a key host factor interacting with the SARS-CoV-2 nucleocapsid (N) protein.	Yes	Both	It potentially enhances SARS-CoV-2 replication.	[93]
	Chikungunya virus (CHIKV)	-	Simultaneous absence of G3BP1 and G3BP2 reduces CHIKV RNA levels, protein expression, and progeny titers.	Yes	Cell line	Chikungunya virus (CHIKV) also relies on G3BPs for efficient replication.	[97]
	Foot-and-mouth disease virus	It can be achieved by targeting the scaffold proteins G3BP1 and G3BP2, and using their L protease to cleave these proteins.	Antagonizing SG formation modulates the global stress response to evade host antiviral defense.	Yes	Cell line	It facilitates the formulation of future control measures against FMDV infection.	[100]
	Dengue virus (DENV)	-	Dengue virus (DENV) RNA binds to P-body (PB)/stress granule (SG) regulatory proteins, including DDX6, G3BP1, G3BP2, Caprin1, and USP10.	Yes	Cell line	DENV-2 3′ UTR acts as an assembly platform for PB/SG-associated proteins; DDX6 assembly on the 3′ UTR is essential for viral replication.	[102]
Cardiovascular and cerebrovascular diseases	Myocardial hypertrophy	Lnc9456 interacts physically with G3BP2.	Promotes NF-κB nuclear translocation.	Yes	Both	This further activates the hypertrophy-related signaling cascade.	[35,107]
		The hypertrophy response of neonatal rat cardiomyocytes (NRCMs) induced by overexpression of G3BP2 or isoproterenol (ISO)	It is significantly inhibited by the NF-κB inhibitor PDTC (50 μmol/L) or p65 knockdown.				
	The microvascular endothelial cells of the human retina	The lncRNA TDRG1 competes for binding with miR-7-5p	Increases G3BP2 transcriptional levels.	Yes	Cell line	strengthens high glucose (HG)-induced damage to human retinal microvascular endothelial cells (hRMECs).	[110]
	Alzheimer’s disease (AD)	-	G3BP2 directly binds to the Tau protein and inhibits its pathological aggregation.	Yes	Cell line	The absence of G3BP2 significantly accelerates the Tau pathological process.	[46,113]
			G3BP2 blocks Tau aggregation by masking its microtubule-binding region (MTBR)				
Other diseases		G3BP2 is involved in immune responses, mRNA transport, and stress granule assembly	-	Yes	Mouse	Mouse spermatogenesis and male fertility are indispensable.	[114]
		The interaction between PGRMC1/PGRMC2 and G3BP2	-	Yes	Cell line	Dynamic regulation of the rate of SIGC cell cycle entry.	[115]
			G3BP2 elevates CD8+ T cell percentage	Yes	Cell line	Promotion of psoriasis development	[116]
		miR-363 is indispensable in the polarization of M1 macrophages	Released from M1 macrophages via exosomes	-	-	Induces chondrocyte apoptosis and inflammation; this axis is a promising target for cartilage degeneration and OA prevention	[117]
			miR-363 inhibits chondrocyte damage by targeting and repressing G3BP2				

## 3. Clinical Treatment Transformation of G3BP2 (Table 2)

The clinical translation of G3BP2 involves two main aspects: its potential utility in disease diagnosis and therapeutic value in disease treatment. In terms of research methods, cell and animal models [118,119], preclinical models, etc., were used to explore the involvement of G3BP2 in the diagnosis and treatment of diseases.

### 3.1. Role of G3BP2 in Clinical Diagnosis

The synergistic interaction between PSF and G3BP2 in the cell nucleus is crucial for preventing aging and the development of AD [120]. Furthermore, G3BP2 binds to NF-κB/p65 by interacting with the NF-κB inhibitor α (IκBα), maintaining its cytoplasmic localization, and restricting its transcriptional activity. Since PGRMC1 and PGRMC2 can bind to G3BP2, when PGRMC1, PGRMC2, or G3BP2 are depleted, the transcriptional activity of NF-κB increases, and cell cycle progression accelerates [121].

### 3.2. Clinical Therapeutic Value of G3BP2

#### 3.2.1. BC

BC is a malignant tumor that originates from epithelial cells of the breast [122]. Currently, the primary treatment methods for BC include surgery, radiotherapy, chemotherapy, endocrine therapy, targeted therapy, and immunotherapy. However, targeted drugs and endocrine therapy are prone to drug resistance and recurrence, while chemotherapy and radiotherapy can cause cardiac toxicity and secondary cancers as side effects. The triple-negative BC lacks specific targets and has limited treatment options. Based on BC pathogenesis, G3BP2 upregulation can promote the migration, stem cell properties, and ptx resistance of BC cells, while downregulation inhibits these properties. These results clarified the key role of G3BP2 in breast cancer progression, providing new therapeutic targets for PTX resistance and breast cancer progression mediated by the circBACH1/miR-217/G3BP2 axis [82].

#### 3.2.2. Head and Neck Squamous Cell Carcinoma

Head and neck squamous cell carcinoma is a malignant tumor that originates from the squamous epithelium of the mucosa in the head and neck region. It commonly occurs in the oral cavity, pharynx, and larynx. More than 90% of head and neck cancers are well-or moderately differentiated squamous cell carcinomas [123]. Currently, the primary treatment methods for this condition include surgery, radiotherapy, chemotherapy, targeted therapy, and immunotherapy. However, there are shortcomings, such as large surgical wounds, limitations of targeted/immune therapy, low early diagnosis rate, and poor prognosis in advanced stages. Based on the pathogenesis of head and neck squamous cell carcinoma, the PRMT5-USP7-G3BP2 regulatory axis participates in tumor development through the mechanism of lipid metabolism reprogramming, providing potential targets for the metabolic treatment of head and neck squamous cell carcinoma [124].

#### 3.2.3. Lung Cancer

Lung cancer is a malignant tumor that originates in the mucosa or glands of the bronchi. It is the most common and fatal malignant tumor worldwide. The primary pathological types of lung cancer are NSCLC and small-cell lung cancer. For stage I or II NSCLC, the primary treatment approach is surgical resection, combined with adjuvant therapy. Chemotherapy or radiotherapy is the primary treatment for stage III or IV cancer. However, traditional chemotherapy drugs generally have problems, such as poor targeting, low bioavailability, and susceptibility to drug resistance, which limit their efficacy [125,126,127]. Based on its pathogenesis, the activity of G3BP2 can control the progression of lung cancer by regulating the TRIM protein family member MG53 (TRIM72) [30].

#### 3.2.4. Pancreatic Ductal Carcinoma

Pancreatic ductal carcinoma is a malignant tumor that originates from the epithelial cells of the pancreatic ducts and accounts for more than 90% of pancreatic cancers. It is characterized by high invasiveness, early metastasis, and poor prognoses. PDAC treatment mainly relies on chemotherapy and surgery. However, 15–20% of patients are suitable for surgery at the time of diagnosis. Most patients have experienced distant metastasis. At this point, resection of the primary tumor is difficult to improve prognosis. Moreover, targeted therapy has made significant progress in overcoming chemotherapy resistance and poor prognosis [128]. Based on their pathogenesis, G3BP2-mediated SGs are protective therapeutic targets for PDAC [72].

#### 3.2.5. PCa

PCa is the second leading cause of cancer-related deaths among men, after lung cancer. Tumors originate from genetic mutations in the epithelial cells of the prostate gland (mostly adenocarcinoma). Prostate biopsy under ultrasound guidance can assist in diagnosis, and androgen deprivation therapy of the gonads is the cornerstone of treatment. The standard treatment plan for localized PCa includes surgery, radiotherapy, and active surveillance [129,130]. However, there are limitations, such as limited early diagnosis, complications of radical treatment, resistance to endocrine therapy, and poor prognosis in advanced stages. Based on its pathogenesis, G3BP2 drives the malignant progression of PCa via two pathways: one is to form a complex with the tumor suppressor p53, inhibiting the regulatory function of the p53 signaling pathway on cell growth. The other is that as its protein increases, it cooperates with the AR signal to promote tumor development jointly. USP10, by upregulating G3BP2 expression, becomes a key molecule with a core oncogenic function in PCa [27].

#### 3.2.6. CRC

CRC is a malignant tumor that originates from the epithelial cells of the colon and rectum, with adenocarcinoma being the predominant type, including colon and rectal cancers. It is a common malignant tumor of the digestive system. Systemic treatment is the primary treatment for CRC. Studies have demonstrated that targeted therapy and immunotherapy can effectively achieve tumor regression and improve survival rates [131]. Research on its pathogenesis has revealed that phosphorylated YBX1 enters the nucleus to activate G3BP2 transcription, triggering the activation of the MAPK pathway. In the AOM/DSS model, Eps8l2 knockout inhibited CRC tumorigenesis. The EPS8L2-YBX1-G3BP2 regulatory axis drives CRC progression and provides a new target for CRC treatment [132].

#### 3.2.7. Oral Cancer

Oral cancer refers to malignant tumors in the mouth, involving areas such as the buccal mucosa and the floor of the mouth. More than 90% of oral cancers originate from squamous tissue, and these types of oral cancer are commonly referred to as oral squamous cell carcinomas. Oral cancer is associated with various factors, including smoking, alcohol consumption, and human papillomavirus [133]. Currently, the primary treatment methods for oral cancer include surgery, radiotherapy, chemotherapy, targeted therapy, and immunotherapy. However, these methods have limitations, such as large surgical wounds, numerous side effects from radiotherapy, and the possibility of drug resistance during chemotherapy. Based on the pathogenesis of oral cancer, G3BP2 has been found to reduce the efficacy of radiotherapy in treating oral cancer [11].

#### 3.2.8. Atherosclerosis

Atherosclerosis is the primary cause of cardiovascular and cerebrovascular diseases. It is a chronic, systemic, inflammatory disease that mainly affects large- and medium-sized arteries. Common risk factors include high blood lipid levels, high blood pressure, smoking, and diabetes. Currently, the incidence, disability, and mortality rates of this disease are relatively high. Moreover, influenced by changes in modern dietary and exercise habits, it is presenting a younger trend [134]. Based on its pathogenesis, in the atherosclerosis model of ApoE^−/−^ mice, immunization with the G3BP2 peptide antigen or knockdown of G3BP2 expression significantly reduced the development of early atherosclerotic plaques. This suggests that G3BP2 is a potential target for treating atherosclerosis [135].

#### 3.2.9. Others

G3BP2 has three mechanisms of action in anti-cancer immunotherapy. First, it drives immunosuppression by regulating the tumor stem cell program and stress response and upregulating immune checkpoint molecules, such as PD-L1, in BC and glioblastoma. Second, it has clinical validation effects, as the co-expression of G3BP2 and PD-L1 in the tumor tissues of patients with cancer is significantly correlated. Third, it has potential for targeted intervention, as the C108 inhibitor or gene inhibition can reduce PD-L1 mRNA expression by promoting its degradation, thereby directly blocking immune escape [80]. Benzopyridine degradation agents can target non-classical CRBN substrates, such as KDM4B, G3BP2, and VCL, all without the CRBN β-hairpin structure. This discovery demonstrates that unbiased proteomics has crucial value in the development of MGD drugs, and the range of new CRBN substrates exceeds the framework of classical immunomodulatory drugs (IMiDs) [136].

**Table 2:** Clinical treatment transformation of G3BP2.

Classification	Disease	Mechanism	Cell Line /Animals	Treatment	Clinical Trial	References
Biomarker	AD	The synergistic interaction of PSF and G3BP2 in the cell nucleus	Animals		Yes	[120]
	-	Depletion of PGRMC1, PGRMC2, or G3BP2 increases NF-κB transcriptional activity.	Animals	The cell cycle progresses at an accelerated pace.	Yes	[121]
Therapy	BC	G3BP2 upregulation enhances breast cancer (BC) cell migration, stemness, and paclitaxel (PTX) resistance; its downregulation abrogates these phenotypes, highlighting a critical role of G3BP2 in BC progression.	Cell line	The circBACH1/miR-217/G3BP2 axis-mediated paclitaxel (PTX) resistance and breast cancer (BC) progression identifies novel therapeutic targets.	Yes	[82]
	Head and neck squamous cell carcinoma	The PRMT5-USP7-G3BP2 regulatory axis drives tumorigenesis via lipid metabolic reprogramming.	Cell line	It identifies potential therapeutic targets for metabolic therapy in head and neck squamous cell carcinoma (HNSCC).	Yes	[124]
	Lung cancer	TRIM72 modulates G3BP2 activity.	-	This suppresses lung cancer progression.		[30]
	PDAC	G3BP2-mediated SGs	-	Protective therapeutic targets for PDAC.		[72]
	PCa	It forms a complex with the tumor suppressor p53, thereby inhibiting the regulation of cell growth by the p53 signaling pathway.	-	USP10 upregulates G3BP2 expression to act as a key mediator of PCa carcinogenesis.		[27]
		Acting synergistically with the AR signaling, they jointly promote tumor development.				
	CRC	Phosphorylated YBX1 translocates to the nucleus to initiate G3BP2 transcription and activate the MAPK signaling pathway. Eps8l2 knockout suppressed CRC tumorigenesis in the AOM/DSS model.	Animals	EPS8L2-YBX1-G3BP2 axis: a driver of CRC progression and a novel therapeutic target.	Yes	[132]
	Oral cancer		-	G3BP2 impairs radiotherapy efficacy in oral cancer.		[11]
	Atherosclerosis	In ApoE−/− atherosclerotic mice, G3BP2 peptide antigen administration or G3BP2 knockdown significantly reduces early atherosclerotic plaques	Animals	It represents a potential therapeutic target for atherosclerosis therapy.	Yes	[135]
	Anti-cancer immunotherapy	Three core findings: immune escape regulation, clinical relevance, targeted therapeutic potential: (1) Driving immunosuppression: G3BP2 modulates tumor initiation (stem cells) and upregulates PD-L1 under stress in BC and glioblastoma, establishing a novel “stem cell program-stress response-immune checkpoint” axis. (2) Clinical relevance validation: G3BP2 and PD-L1 co-expression is significantly correlated in human tumor tissues, supporting their functional association. (3) Targeted therapeutic potential: C108 inhibitor-mediated or genetic inhibition of G3BP2 enhances PD-L1 mRNA degradation and reduces its expression significantly.	-	It identifies a direct target for blocking immune escape.	Yes	[80]
	New substrate of CRBN	Highly selective and potent phenylpyridineamine degraders target uncharacterized novel substrates (KDM4B, G3BP2, VCL), which lack the canonical CRBN β-hairpin motif	-	Marked expansion of the novel CRBN substrate repertoire defined by canonical IMiDs.		[136]

## 4. Summary and Outlook

The function of G3BP2 relies on the characteristics of its variable protein domain, which plays a crucial role in physiological processes by binding to either RNA or proteins. It frequently acts as an upstream signaling regulator, regulating downstream pathways and participating in various cellular functions. It is closely related to diseases such as tumors, inflammation, and viral infections. Inhibiting its activity can block the initiation and progression of diseases, alleviate symptoms, and potentially achieve a long-term cure.

Although preclinical studies have yielded positive results, G3BP2-targeted therapy faces challenges, including target specificity and drug delivery efficiency. However, as a common target for multiple diseases, targeted therapy of G3BP2 is expected to provide novel and precise treatment strategies for tumors, inflammation, and viral infections. In the future, with the development of targeted drugs and optimization of delivery technologies, G3BP2 is expected to become a key therapeutic target in clinical treatment, offering the possibility of a long-term cure for patients.

## Data Availability

This review is based solely on published literature, and no additional primary data were generated or analyzed. All referenced studies are listed in the References section.
